# Awareness of Lebanese Pediatricians regarding Children’s Oral Health

**DOI:** 10.5005/jp-journals-10005-1412

**Published:** 2017-02-27

**Authors:** Nahla Nassif, Balsam Noueiri, Riad Bacho, Kassem Kassak

**Affiliations:** 1 Associate Professor, Department of Pediatric Dentistry, Lebanese University Beirut, Lebanon; 2 Associate Professor, Department of Pediatric Dentistry, Lebanese University Beirut, Lebanon; 3 Associate Professor, Department of Pediatric Dentistry, Lebanese University Beirut, Lebanon; 4 Director and Executive Master, Health Management and Policy Division; Health Care Leadership Program, American University of Beirut, Beirut, Lebanon

**Keywords:** Breastfeeding, Fluoride effectiveness, Fluoride safety, Knoweldge, Oral health, Pediatrician, Primary research.

## Abstract

Pediatricians have an important role in early identification and prevention of oral health, but they reported lack of knowledge in this field.

**Aim:**

the aim of the study is to evaluate the knowledge and attitudes of Lebanese pediatricians regarding children’s oral health.

**Materials and methods:**

A cross sectional study including 100 Lebanese pediatricians was performed. They answered 21 questions. Three variables were taken into consideration: The number of years in practice, the place and type of practice.

**Results:**

73.6% of pediatricians with more than 5 years in practice, 63.5% of pediatricians with an exclusive private practice and 74.7% of pediatricians working in cities/big villages believe that a child is able to brush properly his teeth before the age of 5 years. Only 27.6% of pediatricians with more than 5 years in practice, or working in cities/big villages and 12.7% of those having an exclusive private practice admit that white and black spots are signs of affected teeth.

**Discussion:**

Majority of our pediatricians reported that bottle feeding is associated with early childhood caries. They do not believe that the maternal milk can harm the baby’s teeth. Concerning the transmission from mother to child of the bacteria responsible for dental caries, the reported percentages were not statistically different in relation to pediatricians’ years of experience, type and place of practice.

Pediatricians who are academically affiliated were more likely to report that fluoride is safe compared to those practicing in the private sector (P = 0,012). The majority believe that there is a relation between systematic manifestation such as fever and eruption of primary teeth.

**Conclusion:**

The Lebanese pediatricians have an acceptable level of knowledge in children’s oral health, but should be better informed and motivated toward dental and oral issues.

**How to cite this article:**

Nassif N, Noueiri B, Bacho R, Kassak K. Awareness of Lebanese Pediatricians regarding Children’s Oral Health. Int J Clin Pediatr Dent 2017;10(1):82-88.

## INTRODUCTION

Pediatricians are the primary care providers, and are considered to be the first health encounter for infants during which they are thoroughly examined and necessary health education is provided to parents. Thus, provision of oral health assessment by pediatricians is viewed to be essential for both prevention and early detection of oral conditions.^1^ Usually, dental examination remains rarely included as a part of the medical visits. Dental caries is considered as the most common chronic infectious disease in childhood.^2^

A healthy oral cavity plays a major role in the child’s life through its repercussions on normal nutritional intake, language acquisition, and psychological behavior. Since pediatricians are the first health care providers, they must include the dental exam during the medical visit. They have an important role in early identification and prevention of health in general and dental health in specific. They must be able to give recommendations from the simplest preventive dental treatments till the prevention of early childhood caries (ECC).^3^

Despite the positive attitude that pediatricians show toward the significance of oral health exam and the need to perform it, there is lack of knowledge in relation to essential dental-related facts and practices. Insufficient knowledge related to oral health of children and infants has been reported by pediatricians in several studies.^4,5^ In Lebanon, there have been no studies conducted so far that assess knowledge and attitude of pediatricians in relation to children’s oral health.

The aim of this study is to answer the following research question: What is the prevalent knowledge level and attitudes of pediatricians regarding children’s oral health in Lebanon? In addition to the latter purpose, the objective of this study aims to assess whether the pediatricians’ knowledge and attitudes in oral health differ in terms of their years of experience, place of practice, and type of practice. For this purpose, a questionnaire inspired by previous studies was prepared.^3,6,7^

## MATERIALS AND METHODS

A cross-sectional approach was utilized to conduct this study. Our population of interest was Lebanese pediatricians, who are members of the Lebanese Society of Pediatricians. They were selected from five Governorates -Beirut, Mount Lebanon, North Lebanon, South Lebanon, and Bekaa. The number of doctors from each Governor-ate was proportional to the total number of pediatricians practicing in this Governorate. A total of 100 pediatricians were interviewed at their workplaces (22 doctors from Beirut, 43 doctors from Mount Lebanon, 11 doctors from North Lebanon, 16 doctors from South Lebanon, and 8 doctors from Bekaa).

The questionnaire was adapted to the context of the study, then pretested and modified accordingly. It is divided into two sections, with all questions being close-ended questions. The first section covered pediatrician’s general information and included the following variables: Years of practice, which is divided into less than and more than 5 years; place of practice, which is divided into city/big village and small village; and type of practice, which is divided into either private practice or having an academic affiliation. The second section covered pediatrician’s knowledge and attitude regarding oral health and included nine questions covering the following variables: Age at which children can properly brush their teeth, relation between bottle-/breast-feeding with ECC, transmission of bacteria responsible for dental caries, effectiveness and safety of fluoride intake, signs of affected teeth, relation between primary teeth eruption and systematic manifestations, and familiarity with dental fissure sealants. All questionnaires were completely filled, thus rendering a 100% response rate.

Data were analyzed using Statistical Package for the Social Sciences version 18. Categorical data were analyzed using chi-square test, whereas continuous variables were analyzed using t-test.

## RESULTS

Demographic information of the study revealed that 87% of pediatricians had more than 5 years of experience; 60% had a specialization; 83% worked in cities/big villages, 13% worked in small villages; and 4% worked in both. In relation to the type of practice, 63% of pediatricians practiced in the private sector, 11% had academic affiliation, and 26% had both (Table 1).

Results were analyzed in relation to three aspects: Years of practice, place of practice, and type of practice.

### Knowledge and Attitude Characteristics by Years of Practice

Knowledge and attitude characteristics classified by years of practice of pediatricians revealed that 61.5% of doctors who have been practicing for less than 5 years reported that children can properly brush their teeth after the age of 5 as compared with only 26.4% of those with more experience. Around 28% of pediatricians with more than 5 years of experience reported that both white and black spots are a sign of affected teeth; however, none of those who had less than 5 years of experience considered that both spots are a sign of teeth decay. Around 17% of experienced doctors consider white spots a sign indicating tooth decay, whereas only 7.7% of the less experienced pediatricians consider it a positive sign for decay. Nearly 58% of the pediatricians with more years of experience reported being familiar with dental fissure sealants as compared with only 30.8% among those with less experience.

The majority of the experienced doctors (94.3%) *vs* 84.6% of the less experienced believe that bottle-fed children get ECC. However, only 32.2% of the experienced and 23.1% of the less experienced doctors believed that there is a link between ECC and breastfeeding. Concerning fluoride effectiveness, 88.5 and 92.3% of the experienced and the less experienced group respectively, believe that fluoride is effective in preventing decay. However, a lower number of pediatricians believe that fluoride intake is safe (77% of pediatricians with more than 5 years of experience *vs* 61.5% of pediatricians with less than 5 years of experience). Both pediatricians with more than and less than 5 years of experience (approximately 69% of both groups) believe that there is a relationship between eruption of primary teeth and systematic manifestations. Finally, 64.4% of pediatricians with more than 5 years of experience were more likely to report that the bacteria responsible for causing dental caries is transmitted from mother to child than pediatricians with less than 5 years of experience (46.2%) (Table 2).

### Knowledge and Attitude Characteristics by Type of Practice

Around half of the pediatricians with academic affiliation (43.2%) reported that both black and white spots indicate that teeth are affected compared with 12.7% of pediatricians in private practice. On the contrary, 51.4% of those with academic affiliation compared with 65.1% of those in private practice considered that only black spots are an indication of teeth decay. White spots were reported by 22.2% of doctors in private practice, whereas 5.4% of doctors with academic affiliation indicated that white spots are a sign for teeth decay. Believing that fluoride intake is safe was higher among pediatricians with academic affiliation (89.2%) than those practicing in the private sector (66.7%).

**Table 1:** **Table 1:** Demographic study

*General information*		*Percentage* * of pediatricians*	
*Years of practice*			
Less than 5 years		13	
More than 5 years		87	
*Type of practice*			
Private		63	
Academically affiliated		11	
Both		26	
*Area of practice*			
City/big village		87	
Small village		13	

Almost only 22% of doctors with academic affiliation and 36.5% of those in private practice reported that children can properly brush their teeth after the age of 5 years. More than 90% of doctors in both groups believe that bottle-feeding can result in ECC. A low percentage of both doctors believe that breastfeeding results in ECC (21.6% of those with academic affiliation *vs* 36.5% of those in private practice). Approximately 60% of the doctors in both groups reported that the bacteria responsible for dental caries are transmitted from mother to child. Almost 54% of doctors in both groups reported that they are familiar with dental fissure sealant. Majority of pediatricians with academic affiliation (94.6%) and 85.7% of those in private practice believe that fluoride is effective for preventing decay. Furthermore, both groups reported similar results (67.6% of those with academic affiliation and 69.8% of those in private practice) with respect to believing that there is a relation between eruption of primary teeth and systematic manifestations (Table 3).

**Table 2:** **Table 2:** Knowledge and attitude characteristics by years of practice

				*Years in practice*					
				*More than* * 5 years (%)*		*Less than* * 5 years (%)*		*p-value*	
Age when a child can properly brush teeth		After 5 years		26.4		61.5		0.011	
		Before 5 years		73.6		38.5			
Teeth are affected when they show		White spots		17.2		7.7		0.033	
		Black spots		55.2		92.3			
		Both		27.6		0			
Believe that overnight bottle-fed children gets ECC		Yes		94.3		84.6		0.204	
Believe that overnight breastfeeding is associated with ECC		Yes		32.2		23.1		0.508	
Bacteria responsible for dental caries MTCT		Yes		64.4		46.2		0.207	
Familiar with dental fissure sealants		Yes		57.5		30.8		0.072	
Believe that fluoride is effective in prevention		Yes		88.5		92.3		0.683	
Believe that fluoride is safe in prevention		Yes		77.0		61.5		0.229	
Believe a relationship between eruption of primary teeth and		Yes		69.0		69.2		0.985	
systemic manifestations									

MTCT: Mother-to-child transmission; ECC: Early childhood caries

**Table 3:** **Table 3:** Knowledge and attitude characteristics by type of affiliation

				*Affiliation*					
				*Private practice (%)*		*Affiliation Academic affiliation (%)*		*p-value*	
Age when a child can properly brush his teeth		After 5 years		36.5		21.6		0.120	
		Before 5 years		63.5		78.4			
Teeth are affected when they show		White spots		22.2		5.4		0.001	
		Black spots		65.1		51.4			
		Both		12.7		43.2			
Believe that overnight bottle-fed children get ECC		Yes		92.1		94.6		0.632	
Believe that overnight breastfeeding is associated with ECC		Yes		36.5		21.6		0.120	
Bacteria responsible for dental caries MTCT		Yes		63.5		59.5		0.688	
Familiar with dental fissure sealants		Yes		54.0		54.1		0.993	
Believe that fluoride is effective in prevention		Yes		85.7		94.6		0.171	
Believe that fluoride is safe in prevention		Yes		66.7		89.2		0.012	
Believe that a relationship exists between eruption of		Yes		69.8		67.6		0.812	
primary teeth and systemic manifestations									

MTCT: Mother-to-child transmission; ECC: Early childhood caries

### Knowledge and Attitudes Characteristics by Place of Practice

Pediatricians practicing in small villages were more likely to report that children can properly brush their teeth after the age of 5 (69.2%) compared with pediatricians working in city/big villages (25.3%). Similarly, 26.4% of doctors practicing in the city/big village believed that breastfeeding is associated with ECC, whereas 61.5% of those working in small villages believed that there is an association. Around 28% of doctors working in city/ big village considered that both black and white spots indicated teeth decay, whereas none of those working in small villages indicated the latter.

More than 90% of both groups believed that bottle-feeding results in ECC. Nearly similar percentages were reported by both groups in relation to familiarity with dental fissure sealant (55.2% for those in city/big village *vs* 46.2% for those in small villages). More than half of both groups reported that the bacteria responsible for dental caries are transmitted from mother to child. Majority of both groups believed that fluoride is effective in preventing dental caries. The safety of fluoride intake was reported by 77% of those working in city/ big villages and 61.5% of doctors working in small villages. Finally, 69% of both groups believed that a relation exists between eruption of primary teeth and systematic manifestations (Table 4).

## DISCUSSION

This study has assessed the knowledge level and attitudes of Lebanese pediatricians in relation to children’s oral health. Results were analyzed with respect to years of practice, place of practice, and type of practice of pediatricians. Majority of the study sample (78%) has been practicing pediatrics for more than 5 years, reflecting a good professional experience level, whereas the less experienced who have been working for less than 5 years constitute only 13% of our sample. Majority of pediatricians (63%) work in the private sector and thus, few have teaching activities in local universities. Pediatricians working at teaching institutions are more likely to stay up-to-date in regard to medical information, knowledge, and guidelines that inform the specialty practice. The majority of pediatricians (87%) in our study work in either cities or big villages. It can be justified by the presence of a high density of population in cities, which can lead to a higher number of patients than in small villages.^8^

**Table 4:** **Table 4:** Knowledge and attitude characteristics by place of practice

				*Practice area*					
				*City/big village (%)*		*Small village (%)*		*p-value*	
Age when a child can properly brush their teeth		After 5 years		25.3		69.2		0.001	
		Before 5 years		74.7		30.8			
Teeth are affected when they show		White spots		13.8		30.8		0.054	
		Black spots		58.6		69.2			
		Both		27.6		0			
Believe that overnight bottle-fed children get ECC		Yes		93.1		92.3		0.916	
Believe that overnight breastfeeding is associated with ECC		Yes		26.4		61.5		0.011	
Bacteria responsible for dental caries MTCT		Yes		63.2		53.8		0.516	
Familiar with dental fissure sealants		Yes		55.2		46.2		0.543	
Believe that fluoride is effective in prevention		Yes		88.5		92.3		0.683	
Believe that fluoride is safe in prevention		Yes		77.0		61.5		0.229	
Believe that a relationship exists between eruption of primary		Yes		69.0		69.2		0.985	
teeth and systemic manifestations									

MTCT: Mother-to-child transmission; ECG: Early childhood caries

According to the American Academy of Pediatricians (AAP), the child can properly brush his/her teeth around the age of 8 where he/she will be able to practice this skill without assistance.^9^ In our study, pediatricians with less than 5 years of experience (61.5%) and those working in small villages (69.2%) were more likely to report the age at which a child can properly brush his/her teeth. The former result can be explained by the ability of newly graduating pediatricians to better recall information taught during medical school years and by the fact that new medical graduates are more likely nowadays to utilize evidence-based research.

Pediatricians with more than 5 years of experience, with academic affiliation and working in city/big villages, were more likely to report that both black and white spots indicate a sign of tooth decay, with the highest percentage reported in those having an academic affiliation (43.2%, p = 0.001). None of pediatricians with less than 5 years of experience and working in small villages reported that both black and white spots are signs of affected teeth (p = 0.003 and p = 0.054 respectively). White spot lesions are the earliest stage of tooth decay and are most commonly seen on the visible “facial” surfaces of teeth as frosty white areas.^10^ Prakash et al^11^ noticed that 46% of pediatricians and 62% of family physicians were not sure that white spots or lines on tooth surfaces were the first signs of tooth decay. This lack of knowledge was explained by Lewis et al^1^ who found that less than 25% of pediatricians had received oral health education in medical schools. Generally, pediatricians and physicians indicated the need for increasing their knowledge through continual education courses.^12^ This can be a serious issue knowing that the child will not be referred to the dentist.

Majority of our pediatricians regardless of their years of experience, place, and type of practice reported that overnight bottle-feeding is associated with ECC. A similar result was reported by Prakash et al.^11^ However, only pediatricians who are practicing in small villages have associated ECC with overnight breastfeeding (61.5%) compared with just 26.4% of those working in cities and big villages (p = 0.011). Years of experience and type of practice were not seen to be associated with knowledge about breastfeeding and ECC risk;^13^ however, the majority in these two groups did not believe that breastfeeding and dental caries are linked. The later result is comparable with a study conducted in Brazil, which found that around half of pediatricians regardless of their years of experience did not believe that there is a link between overnight breastfeeding and ECC.^4^ Lack of knowledge regarding this aspect might be linked to the dominant misbelief that breast milk does not cause dental caries.^13^ Bowen and Lawrence^14^ reported that even though human milk is more cariogenic than cow milk, it is less cariogenic than formula milk. Accordingly, the AAP recommends breastfeeding for 9 months and limits it to two times per day beyond the age of 1.^9^

As known, *Mutans streptococci* is the most important bacteria responsible for dental caries. This bacteria can be transmitted from mother to child.^15^ Microbiological studies have proved that children usually attain *S. mutans* from their mothers, who are the main source of transmission of dental caries. Based on a systematic review and meta-analysis, Da Silva Bastos et al^16^ concluded that there is scientific evidence of mother-to-child *S. mutans* transmission The American Academy of Pediatric Dentistry (AAPD) has recommended avoiding saliva-sharing behaviors between mothers and their infants, such as sharing eating utensils and pacifiers. It has also recommended preserving maternal dental health through providing oral health education and performing regular dental assessments during and after pregnancy to reduce the risk of bacteria colonization and thus transmission to infants.^17^

In our study, between 46.2 and 64.4% of pediatricians reported that bacteria responsible for dental caries can be transmitted from mothers to children. The reported percentages were not statistically different in relation to pediatricians’ years of experience, type, and place of practice. Results similar to our study have been reported by Prakash et al^11^ where around 24% of pediatricians disagreed or strongly disagreed and nearly 53% were not sure that dental caries can spread from mothers to their babies. Similarly, only 44.7% of pediatricians believed that bacteria responsible for dental caries can get transmitted.^3^ Pediatricians should educate the parents, especially the mother, about the misconception that the mother’s saliva cannot affect the child’s oral health.

Around only half of the pediatricians reported being familiar with dental fissure sealants; however, no statistically significant difference has been found in relation to years, type, and place of practice of pediatricians. Sandalli et al^3^ showed that only 15% of pediatricians are familiar with sealants, and as little as 7% can explain the reason behind sealant application to primary caregivers. Sealant application is considered a prevention measure to reduce the risk for dental caries,^9,18^ especially among children and adolescents who are at higher risk for caries.^19^ Insufficient knowledge and lack of the guidelines for the use of fissure sealant by the pediatricians lead to low recommendations.

The use of fluoride has been recommended by the AAPD to reduce the risk of caries. The AAPD recommends the use of fluoride as a safe and effective measure that needs to be tailored based on a risk and needs assessment of individual children to avoid the risk of fluorosis.^17^ Several studies reported effectiveness of various forms of fluoride products (e.g., varnish, fissure sealants, mouth rinse, toothpaste, etc.).^20,21^ In our study, although the majority believed that fluoride is effective, however, differences were reported in regard to fluoride safety. Pediatricians who are academically affiliated were more likely to report that fluoride is safe compared with those practicing in the private sector (p = 0.012). Knowledge deficiency in relation to fluoride and its use among pediatricians was reported in several studies.^1,3-5^

Majority of our pediatricians believe that there is a relation between systematic manifestation, such as fever and eruption of primary teeth, with no statistical difference in relation to years of experience, place, and type of practice. Shapira et al^22^ found that there are increased levels of the inflammatory cytokines interleukin (IL)-1(3, IL-8, and tumor necrosis factor alpha in the gingival cre-vicular fluid of erupting primary teeth. This increase could explain such clinical manifestations as fever, diarrhea, increased crying, and sleeping and eating disturbances that occur at this time.^22^ Ramos-Jorge et al^23^ noticed that some manifestations, such as irritation, decreased appetite, runny nose, and increased salivation were found to be significantly associated with teething. However, Soares et al^4^ reported that 74.7% did not link systematic symptoms with teething. Ramos-Jorge et al^23^ and Memarpour et al^24^ concluded that there is no link between generalized body symptoms like fever and primary teeth eruption.

## CONCLUSION

In general, our study shows that pediatricians in Lebanon have an acceptable level of knowledge and attitude in relation to children’s oral health.

Pediatricians should be better informed about the age at which children can properly brush their teeth, identifying early signs of dental caries by recognizing white lesions as an early sign of teeth decay, the association between breastfeeding and dental caries, importance of dental fissure sealants, and the absence of association between teething and systematic symptoms. Further attention is also required to reinforce information and knowledge in relation to the risk of bacteria transmission from mothers to their babies and the safety of fluoride in preventing dental caries. The latter results shed light on the significance of assessing the level of education and training of oral health education received during medical school years and residency period. Nevertheless, abiding by oral health updated guidelines that are adopted by the Lebanese Society of Pediatricians should, in turn, be reinforced and maintained through incorporating oral health education into the continuing medical education process.

Since children are exposed to medical care at an early age, primary care medical providers must have the proper knowledge and attitude allowing them to play an important role in helping children and their families to access dental care. This study reveals several points that require immediate attention in which pediatricians should be better informed about oral issues. Nevertheless, abiding by oral health updated guidelines that are adopted by the Lebanese Pediatrician Society should, in turn, be reinforced and maintained through incorporating oral health education into the continuing medical education process. Moreover, the interface between medical and dental practitioners has to be improved by more scientific meetings.

## References

[1] Lewis CW, Boulter S, Keels MA, Krol DM, Mouradian WE, O’Connor KG, Quinonez RB (2009). Oral health and pediatricians: results of a national survey.. Acad Pediatr.

[2] Silk H (2010). Making oral health a priority in your preventive pediatric visits.. Clin Pediatr.

[3] Sandalli N, Kuvvetli SS, Cildir SK, Ergeneli S (2007). The pediatricians’ role in the oral health of children.. OHDMBSC.

[4] Soares IMV, da Silva AMRB, Moura LdFAd, Lima MdDMd, Nétto S, Moura MSd (2013). Conduct of pediatricians in relation to the oral health of children.. Rev Odontol UNESP.

[5] Balaban R, Aguiar CM, Da Silva Araujo AC, Dias Filho EBR (2012). Knowledge of paediatricians regarding child oral health.. Int J Paediatr Dent.

[6] Bhat PK, Aruna C, Badiyani BK, Alle R (2014). Knowledge and attitude on infant oral health among graduating medical students in Bangalore City, India.. JIMSA.

[7] Nammalwar RB, Rangeeth P (2012). Knowledge and attitude of pediatricians and family physicians in Chennai on pediatric dentistry: a survey.. Dent Res J.

[8] Probst J, Moore C, Baxley E, Lammie J (2002). Rural-urban differences in visits to primary care physicians.. Fam Med.

[9] (2014). American Academy of Pediatricians. Updates Schedule of Screening and Assessment for well-child visit..

[10] Cochrane N, Cai F, Huq N, Burrow M, Reynolds E (2010). New approaches to enhanced remineralization of tooth enamel.. J Dent Res.

[11] Prakash P, Lawrence HP, Harvey BJ, McIsaac WJ, Limeback H, Leake JL (2006). Early childhood caries and infant oral health: paediatricians’ and family physicians’ knowledge, practices and training.. Paediatr Child Health.

[12] Sánchez OM, Childers NK, Fox L, Bradley E (1997). Physicians’ views on pediatric preventive dental care.. Pediatr Dent.

[13] Paglia L (2015). Does breastfeeding increase risk of early childhood caries?. Eur J Paediatr Dent.

[14] Bowen WH, Lawrence RA (2005). Comparison of the cariogenicity of cola, honey, cow milk, human milk, and sucrose.. Pediatrics.

[15] Marrs J, Trumbley S, Malik G (2011). Early childhood caries: determining the risk factors and assessing the prevention strategies for nursing intervention.. Pediatr Nurs.

[16] Bastos V, Freitas-Fernandes L, Fidalgo T, Martins C, Mattos C, de Souza I, Mala LC (2015). Mother-to-child transmission of Streptococcus mutans: a systematic review and meta-analysis.. J Dent.

[17] American Academy of Pediatric Dentistry. Guideline on Infant Oral Health Care. 2014 ;37(6):15-16. Available from:. http://www.aapd.org/media/policies_guidelines/g_infantoral-healthcare.pdf.

[18] Azarpazhooh A, Main P (2008). Pit and fissure sealants in the prevention of dental caries in children and adolescents: a systematic review.. J Can Dent Assoc.

[19] Ahovuo-Saloranta A, Forss H, Hiiri A, Nordblad A, Makela M (2016). Pit and fissure sealants versus fluoride varnishes for preventing dental decay in the permanent teeth of children and adolescents.. Cochrane Database Syst Rev.

[20] Jagan P, Fareed N, Battur H, Khanagar S, Bhat M, Basapathy R (2015). Effectiveness of sodium fluoride mouthrinses on the prevention of dental caries: a systematic review.. J Indian Assoc Public Health Dent.

[21] Takeshita E, Danelon M, Castro L, Sassaki K, Delbem A (2015). Effectiveness of a toothpaste with low fluoride content combined with trimetaphosphate on dental biofilm and enamel demineralization *in situ.*. Caries Res.

[22] Shapira J, Berenstein-Ajzman G, Engelhard D, Cahan S, Kalickman I, Barak V (2003). Cytokine levels in gingival crevicular fluid of erupting primary teeth correlated with systemic disturbances accompanying teething.. Pediatr Dent.

[23] Ramos-Jorge J, Pordeus I, Ramos-Jorge M, Paiva S, Isabela A (2011). Prospective longitudinal study of signs and symptoms associated with primary tooth eruption.. Pediatrics.

[24] Memarpour M, Soltanimehr E, Eskandarian T (2015). Signs and symptoms associated with primary tooth eruption: a clinical trial of nonpharmacological remedies.. BMC Oral Health.

